# Supplementary material for: Peeking into the Stingers: A Comprehensive SWATH-MS Study of the European Hornet Vespa crabro (Linnaeus, 1758) (Hymenoptera: Vespidae) Venom Sac Extracts
Source: Int J Mol Sci. 2024 Mar 28;25(7):3798. doi: 10.3390/ijms25073798 (PMC11011553; doi:10.3390/ijms25073798)
Supplement: Supplementary file 1 [file ijms-25-03798-s001.zip › Supplementary Figures S1, S2 and S3.pdf]

Supplementary Material: Peeking into the Stingers: A Comprehensive SWATH-MS Study of the European Hornet *Vespa crabro* (Linnaeus, 1758) (Hymenoptera: Vespidae) Venom Sac Extracts

Xesús Feás, Manuela Alonso-Sampedro, Susana Belén Bravo and Carmen Vidal

Detailed description of the: (1) sampling area; (2) insect localization and identification; and (3) nest removal.

1. Sampling Area

Field research aimed at collecting the *Vespa crabro* insects was conducted in the Galician municipality of Pazos de Borbén. Galicia is located in the northwest of Europe on the Iberian Peninsula (Figure S1).

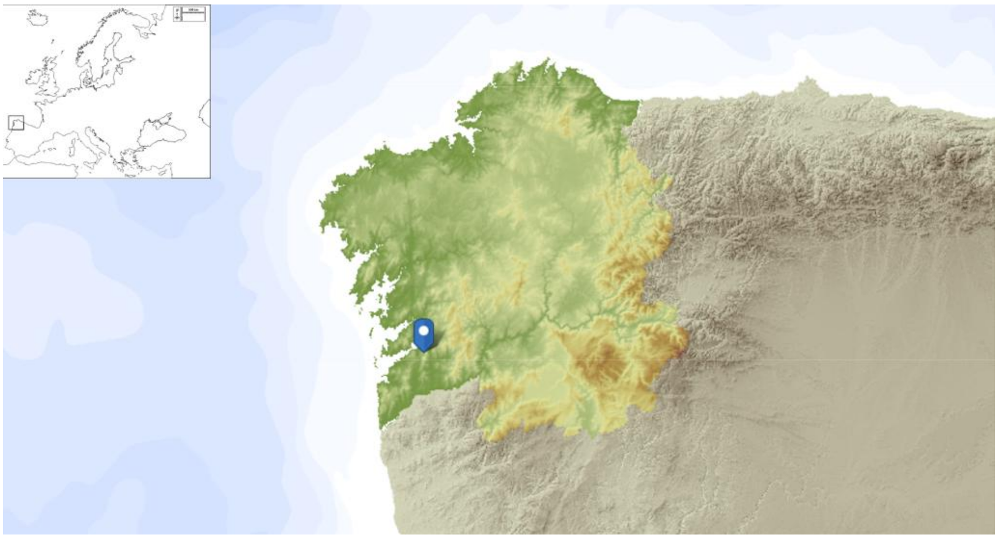

Figure S1. The nest of the European hornet *Vespa crabro* (Linnaeus, 1758) (Hymenoptera: Vespidae) was collected in Pazos de Borbén (coordinates: 42.3451657, -8.4453559), depicted in blue on the hypsometric map of Galicia.

2. Insect Localization and Identification

Eucalyptus logging points were identified within the study area and strategically selected as potential hotspots for vespid activity (Figure S2). The identification of *Vespa crabro* individuals was conducted based on their external morphological characteristics. Easily distinguished from V. *velutina* by coloration (Figure S2b), in particular, the coloration of the second

abdomen tergum (which has the posterior half almost entirely yellow, with the part clearly tridentate apically), and that of the fifth and sixth tergs (which are almost entirely yellow coloring.

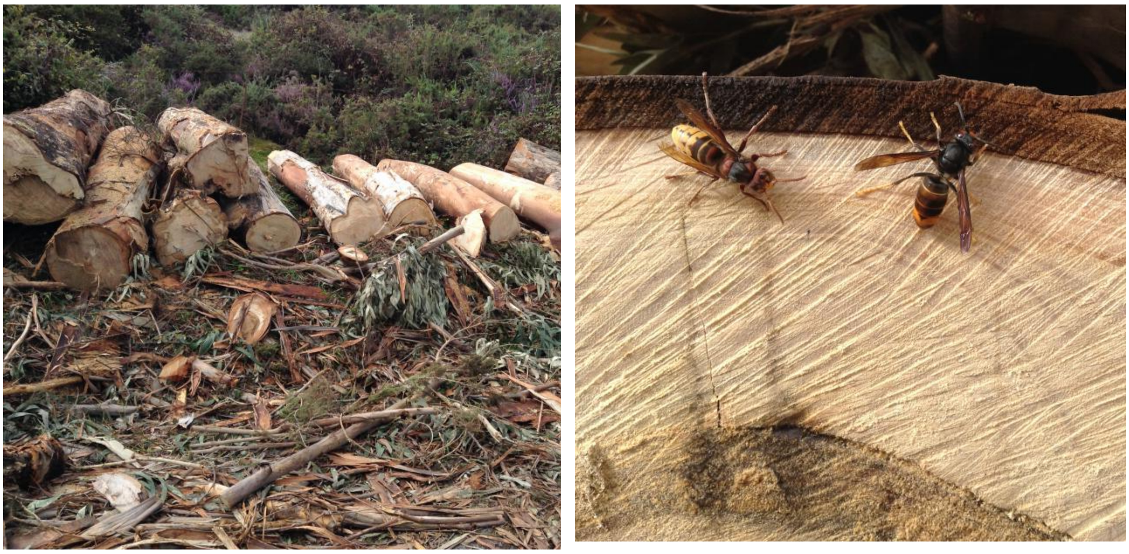

(**a**) (**b**)

Figure S2. (**a**) An *Eucalyptus* logging point; (**b**) Detail of a specimen of European hornet *Vespa crabro* (Linnaeus, 1758) (left) and *Vespa velutina* (Lepeletier, 1836) (right) on cut eucalyptus trunks. Photo Author: X. Feás.

3. Nest removal

The nest of the European hornet, *Vespa crabro* was collected on 5 December 2022 during the night (Figure S3). A complete apiary suit designed to offer full body protection was utilized during the nest collection activity. For the removal of the nest, firstly, its lower opening was covered to contain the insects inside. Subsequently, with the help of a spatula, its adherent parts were separated from the wall. It was then placed in carton box for 24 hours at a temperature of −18 °C in a freezer, until it is taken to the laboratory. The nest was affixed to the wall within a firewood storage at a height of 2 m above the ground, distinguishing: a very friable outer layer of paper-like material, typically in varying shades of grey and brown, that is wrapped around, except for a small opening at the bottom and an internal chamber, with eight combs.

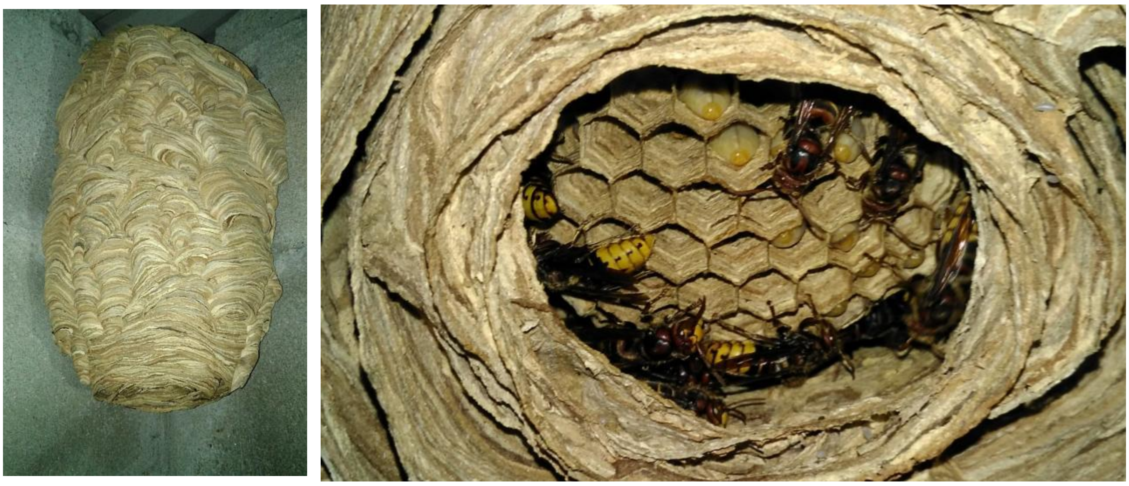

(**a**) (**b**)

Figure S3. The nest of the European Hornet *Vespa crabro* (Linnaeus, 1758) (Hymenoptera: Vespidae) found and subsequently removed (**a**) A side view; (**b**) a bottom view showing its opening. Photo Author: D. Slizt.
