# Supplementary material for: Peeking into the Stingers: A Comprehensive SWATH-MS Study of the European Hornet Vespa crabro (Linnaeus, 1758) (Hymenoptera: Vespidae) Venom Sac Extracts
Source: Int J Mol Sci. 2024 Mar 28;25(7):3798. doi: 10.3390/ijms25073798 (PMC11011553; doi:10.3390/ijms25073798)
Supplement: Supplementary file 1 [file ijms-25-03798-s001.zip › Supplementary Tables S1 and S2.pdf]

Peeking into the Stingers: A Comprehensive SWATH-MS Study of the European Hornet *Vespa crabro* (Linnaeus, 1758) (Hymenoptera: Vespidae) Venom Sac Extracts

Xesús Feás, Manuela Alonso-Sampedro, Susana Belén Bravo and Carmen Vidal

Table S1. Proteins from class Insecta with upregulated expression in *Vespa crabro* gynes venom sac extract.

Protein	Uniprot code	Species	P Value (*t* test)	FC (VSE-G/VSE-W)
**Glycogen debranching enzyme family**				
Glycogen debranching enzyme	A0A7M7M151	*Apis mellifera*	0.004	40.141
**Hexokinase family**				
Phosphotransferase	A0A6P5HFS8	*Bombus terrestris*	< 0.001	15.077
	A0A154PKM2	*Dufourea novaeangliae*	0.004	2.744
**Class I fructose-bisphosphate aldolase family**				
Fructose-bisphosphate aldolase	V9IF40	*Apis cerana*	< 0.001	12.933
**Calponin family**				
Transgelin	A0A7M7GX24	*Apis mellifera*	0.006	12.766
	A0A6P3UBY6	*Bombus terrestris*	< 0.001	4.731
**Glycogen phosphorylase family**				
Alpha-1,4 glucan phosphorylase	A0A6P5I5Q8	*Bombus terrestris*	< 0.001	10.623
	A0A154PP88	*Dufourea novaeangliae*	< 0.001	10.014
	A0A7M7GQ45	*Apis mellifera*	0.001	6.047
**Actin family**				
Actin, indirect flight muscle	V9IG73	*Apis cerana*	0.028	10.101
actin-interacting protein 1 isoform X1	A0A6P3DB16	*Bombus terrestris*	0.004	4.993
**Heat shock protein family**				
heat shock protein beta-1 isoform X2	A0A6P3DLV5	*Bombus terrestris*	< 0.001	9.777
heat shock 70 kDa protein 4 isoform X2	A0A6P3UJ18	*Bombus terrestris*	0.018	1.761
**Protein kinase superfamily, CAMK Ser/Thr protein kinase family**				
Titin	A0A833W593	*Frieseomelitta varia*	< 0.001	7.688

twitchin isoform X2	A0A6P5I2Y9	*Bombus terrestris*	0.003	12.711
Twitchin	A0A154PIM3	*Dufourea novaeangliae*	0.002	2.201
**WD repeat EIF2A family**				
Eukaryotic translation initiation factor 2A	A0A7M7RBB8	*Apis mellifera*	< 0.001	7.264
**GPI family**				
Glucose-6-phosphate isomerase	R9QKY2	*Bombus humilis*	0.042	6.371
**ATPase alpha/beta chains family**				
ATP synthase subunit beta	A0A833VWR2	*Frieseomelitta varia*	0.016	5.725
ATP synthase subunit alpha	V9IFD6	*Apis cerana*	0.046	2.512
**Alpha-ketoglutarate dehydrogenase family**				
oxoglutarate dehydrogenase	A0A154P1T4	*Dufourea novaeangliae*	0.044	5.271
**Citrate synthase family**				
Citrate synthase	A0A154PEK6	*Dufourea novaeangliae*	< 0.001	5.199
**6-phosphogluconate dehydrogenase family**				
6-phosphogluconate dehydrogenase, decarboxylating	A0A833W8Q7	*Frieseomelitta varia*	0.041	4.594
**Malic enzymes family**				
Malic enzyme	A0A154PKU8	*Dufourea novaeangliae*	< 0.001	4.534
	A0A6P3DJM2	*Bombus terrestris*	0.013	2.787
	A0A833S1K4	*Frieseomelitta varia*	0.016	2.013
**Phosphohexose mutase family**				
phosphoglucomutase (alpha-D-glucose-1,6-bisphosphate-dependent)	A0A154PH97	*Dufourea novaeangliae*	0.005	4.135
**Class-I pyridoxal-phosphate-dependent aminotransferase family**				
alanine transaminase	V9IKQ3	*Apis cerana*	< 0.001	3.878
**Cation transport ATPase (P-type) (TC 3.A.3) family**				
Calcium-transporting ATPase	A0A833WFE3	*Frieseomelitta varia*	0.025	3.694
**ATPase alpha/beta chains family**				
H(+)-transporting two-sector ATPase	A0A833W0Z5	*Frieseomelitta varia*	0.016	3.562
**Classic translation factor GTPase family**				
Elongation factor 1-alpha	A0A154P1Q6	*Dufourea novaeangliae*	0.026	3.388
**Transaldolase family**				
Transaldolase	A0A154P208	*Dufourea novaeangliae*	< 0.001	3.150
**Pyruvate kinase family**				
Pyruvate kinase	A0A154PSS0	*Dufourea novaeangliae*	0.001	3.112

**MDH type 1 family**				
Malate dehydrogenase	A0A154PN63	*Dufourea novaeangliae*	0.007	3.004
**Actin-binding proteins ADF family**				
Cofilin/actin-depolymerizing factor	V9II66	*Apis cerana*	< 0.001	2.025
**Peptidase M1 family**				
Aminopeptidase	A0A833S8G1	*Frieseomelitta varia*	0.007	1.924
**Succinate/malate CoA ligase beta subunit family**				
Succinate--CoA ligase [ADP-forming] subunit beta, mitochondrial	A0A833SBT7	*Frieseomelitta varia*	0.044	1.901
**No family described**				
UTP--glucose-1-phosphate uridylyltransferase	A0A6P3UDI8	*Bombus terrestris*	0.021	2.284
Stress-induced-phosphoprotein 1	V9IJY9	*Apis cerana*	0.004	3.752
Phosphoglycerate kinase	A0A6P3U6Z7	*Bombus terrestris*	0.045	2.293
Myosin light chain alkali	V9IF54	*Apis cerana*	0.039	30.289
muscle LIM protein Mlp84B isoform X2	A0A7M7TFB5	*Apis mellifera*	0.016	34.597
Multifunctional fusion protein	A0A6P3DM36	*Bombus terrestris*	0.049	2.005
LOW QUALITY PROTEIN: protein dj-1beta	A0A6P3DG00	*Bombus terrestris*	0.001	4.927
lamin-C isoform X2	A0A6P3U6Y5	*Bombus terrestris*	0.001	3.327
Lactoylglutathione lyase	A0A6P3UJ80	*Bombus terrestris*	0.006	2.104
Glyceraldehyde-3-phosphate dehydrogenase	A0A6P3DDE1	*Bombus terrestris*	0.002	10.925
Glutathione S-transferase	V9IJF3	*Apis cerana*	< 0.001	7.419
Amphiphysin	V9ILS0	*Apis cerana*	0.001	4.341
alpha-actinin, sarcomeric isoform X1	A0A6P3U6D2	*Bombus terrestris*	0.001	5.621
aldose reductase	A0A7M7RB96	*Apis mellifera*	0.001	4.080
1,4-alpha-glucan branching enzyme	A0A6P3TVX5	*Bombus terrestris*	0.031	9.448
Vitellogenin	G8IIT0	*Vespula vulgaris*	< 0.001	17.755
troponin T, skeletal muscle isoform X3	A0A6P5I4V8	*Bombus terrestris*	0.004	6.312
troponin C type I isoform X3	A0A7M7GV60	*Apis mellifera*	0.033	2.457
talin-1 isoform X1	A0A7M7SRL2	*Apis mellifera*	0.038	1.779
LIM domain-binding protein	A0A2A3EBK6	*Apis cerana cerana*	0.007	2.388
Nidogen	A0A833W2G7	*Frieseomelitta varia*	0.007	2.081
Chemosensory protein	Q5Q049	*Vespa crabro*	< 0.001	3.476
peptidylprolyl isomerase	V9IL23	*Apis cerana*	0.041	1.594

VSE-G, *Vespa crabro* gyne venom sac extract; VSE-W, *Vespa crabro* worker venom sac extract; FC (VSE-G/VSE-W), fold change of gyne/worker; FC > 1, upregulated protein in VSE-G; Statistical differences by *t* Test, $p < 0.05$.

Table S2. Proteins from class Insecta with downregulated expression in *Vespa crabro* gynes venom sac extract.

Protein	Uniprot code	Species	P Value (*t* test)	FC (VSE-G/VSE-W)
**CRISP family**				
Venom allergen 5	P0DMB9	*Vespa velutina*	0.006	0.026
**Lipase family**				
Phospholipase A1	C0HLL3	*Vespa velutina*	< 0.001	0.052
Phospholipase A1 verutoxin-2b	P0DMB8	*Vespa velutina*	0.041	0.089
**Peroxiredoxin family**				
Peroxiredoxin-6	V9IET2	*Apis cerana*	0.042	0.321
**2-oxoacid dehydrogenase family**				
Dihydrolipoamide acetyltransferase component of pyruvate dehydrogenase complex	A0A7M7R8J0	*Apis mellifera*	0.012	0.125
**MAM33 family**				
Ras-related protein Rab-43	A0A833W2V6	*Frieseomelitta varia*	0.011	0.136
**Prohibitin family**				
Prohibitin	V9IIF6	*Apis cerana*	0.412	0.148
**Glycosyl hydrolase family**				
Hyaluronidase A	C0HLL4	*Vespa velutina*	0.001	0.148
Hyaluronidase B	C0HLL5	*Vespa velutina*	< 0.001	0.280
Alpha-galactosidase	A0A7M7H421	*Apis mellifera*	0.001	0.390
**Peptidase family**				
Aminopeptidase	A0A154PMQ1	*Dufourea novaeangliae*	0.021	0.186
	A0A833RCD6	*Frieseomelitta varia*	0.013	0.325
Proteasome subunit alpha type	A0A833W9A3	*Frieseomelitta varia*	< 0.001	0.313
**Cu-Zn superoxide dismutase family**				
Superoxide dismutase [Cu-Zn]	A0A7M7MMK3	*Apis mellifera*	< 0.001	0.205
**Aldehyde dehydrogenase family**				
Retinal dehydrogenase	A0A2A3EJU2	*Apis cerana cerana*	0.028	0.257
**Glyceraldehyde-3-phosphate dehydrogenase family**				
Glyceraldehyde-3-phosphate dehydrogenase	A0A088AHC8	*Apis mellifera*	< 0.001	0.274
**NAD-dependent glycerol-3-phosphate dehydrogenase family**				

Glycerol-3-phosphate dehydrogenase [NAD(+)]	A0A154PH44	*Dufourea novaeangliae*	0.022	0.308
**Triosephosphate isomerase family**				
Triosephosphate isomerase	A4ZXC4	*Apis mellifera*	0.016	0.282
**Class-I pyridine nucleotide-disulfide oxidoreductase family**				
Dihydrolipoyl dehydrogenase	A0A7M7R758	*Apis mellifera*	0.034	0.369
**Heat shock protein family**				
Heat shock protein 83	A0A154PI85	*Dufourea novaeangliae*	0.047	0.604
**No family described**				
carbonyl reductase (NADPH)	A0A6P3DFV2	*Bombus terrestris*	0.047	0.193
Chemosensory protein 1	A0A384ZVP9	*Vespa velutina*	0.001	0.302
Calcium-transporting ATPase	A0A6P5I2P0	*Bombus terrestris*	0.030	0.249
Hornet silk protein Vssilk 4	D6RU88	*Vespa xanthoptera*	0.032	0.312
Hornet silk protein Vssilk 3	A9CMG8	*Vespa xanthoptera*	0.033	0.454
Dipeptidylpeptidase IV	A4UA14	*Vespa basalis*	0.015	0.216
Anticoagulant serine protease	B7SD94	*Vespa magnifica*	< 0.001	0.150
Calmodulin	V9I7W9	*Apis cerana*	0.025	0.052
alpha-glucosidase	L7ZBB3	*Apis cerana indica*	< 0.001	0.066
	Q25BT7	*Apis mellifera*	0.005	0.225
	A0A6P3DPJ9	*Bombus terrestris*	0.037	0.301
uncharacterized protein LOC100642610	A0A6P3U365	*Bombus terrestris*	0.025	0.114

VSE-G, *Vespa crabro* gyne venom sac extract; VSE-W, *Vespa crabro* worker venom sac extract; FC (VSE-G/VSE-W), fold change of gyne/worker; FC > 1, upregulated protein in VSE-G; Statistical differences by *t* Test, $p < 0.05$.
